# Ciliochoroidal Detachment After Intrascleral Lens Fixation Using the Yamane Technique

**DOI:** 10.7759/cureus.66562

**Published:** 2024-08-10

**Authors:** Ayako Sadahide, Hiromi Ohara, Ryoya Oda, Yosuke Harada

**Affiliations:** 1 Department of Ophthalmology and Visual Science, Graduate School of Biomedical Sciences, Hiroshima University, Hiroshima, JPN; 2 Mathematics Program, Graduate School of Advanced Science and Engineering, Hiroshima University, Hiroshima, JPN

**Keywords:** vitrectomy, intraocular pressure, intrascleral lens fixation, ciliochoroidal detachment, yamane technique

## Abstract

Purpose: The purpose of this study was to compare the incidence of ciliochoroidal detachment (CCD) after intrascleral lens fixation using the Yamane technique and other vitrectomy procedures.

Methods: This retrospective study evaluated patients who underwent intrascleral lens fixation using the Yamane technique at Hiroshima University Hospital between March 2023 and February 2024 and who could be followed up for at least one month. Patients who underwent vitrectomy for macular disease without air-fluid exchange comprised the control group. The frequency of CCD was compared using anterior segment optical coherence tomography imaging.

Results: Forty-five eyes of 45 patients (26 men and 19 women, mean age 70.8 years) were included. There were no significant differences in the population means or proportions between the intrascleral fixation and control groups for age, sex ratio, right-to-left eye ratio, preoperative visual acuity, preoperative intraocular pressure (IOP), ocular axis, and corneal thickness. The population mean of IOP on the day after surgery was significantly lower in the Yamane intrascleral fixation group (8.4 mmHg) than in the control group (11.5 mmHg) (P < 0.05). There was no significant difference in the population proportions of CCD on the day after surgery between the Yamane intrascleral fixation group and the control group. However, the CCD incidence was 20 eyes (80%) for the Yamane intrascleral fixation group and 12 eyes (60%) for the control group, which was higher in the intrascleral fixation group. There was no significant difference in population means of IOP or population proportions of CCD at one week and one month.

Conclusions: There was no significant difference in population proportions of CCD on the day after surgery, although the CCD rate for the Yamane intrascleral fixation group was higher, and the population mean of the IOP was significantly lower. The Yamane technique assumedly lowered IOP because of the stress placed on the ciliary body. One week after the procedure, the IOP in the intrascleral fixation group normalized.

## Introduction

Gabor and Pavlidis first reported a scleral fixation intraocular lens (IOL) procedure without sutures for IOL implantation in an aphakic eye lacking capsular support [1]. Subsequently, owing to the report "Flanged Intraocular Lens Fixation with Double-Needle Technique" by Yamane et al. in 2017, the Yamane technique for intrascleral fixation is now used at numerous institutions. Compared to scleral suture fixation, intrascleral fixation has reduced postoperative complications such as suture erosion, suture irritation, and re-dislocation due to suture breakage [2]. However, postoperative complications still exist [3-5]. Yamane et al. reported that five eyes (5%) had vitreous hemorrhage, two eyes (2%) had low intraocular pressure (IOP), and two eyes (2%) had elevated IOP as early complications of double-needle intrascleral fixation [2]. In a previous report from our institution on intrascleral fixation, a low postoperative IOP was observed in 10 of 40 eyes [6]. We also encountered a case of hypotony with ciliochoroidal detachment (CCD) and macular hypotony that persisted for three months following the Yamane technique for intrascleral lens fixation. Ciliary suturing was required to improve hypotony.

Previous reports on CCD as an early complication after the Yamane technique of intrascleral fixation are limited to case reports. At our institute, the Yamane technique is combined with pars plana vitrectomy, resulting in two additional scleral incision sites in addition to the incision for pars plana vitrectomy. Pars plana vitrectomy itself has been reported to cause CCD postoperatively, suggesting that the Yamane technique may theoretically increase the risk of CCD. The accumulation of CCD data following the Yamane technique to validate the safety of this surgical method is needed. In this study, we used anterior segment optical coherence tomography (AS-OCT) to compare the frequency of CCD after intrascleral lens fixation using the Yamane technique with other vitrectomy procedures.

## Materials and methods

Patients

This retrospective study evaluated patients who underwent the Yamane technique for intraocular lens fixation at Hiroshima University Hospital between March 2023 and February 2024. To compare the rate of postoperative CCD, we included vitreoretinal disease cases, such as epiretinal membrane (ERM), vitreomacular traction syndrome (VMTS), and stage 1 macular hole (MH), that underwent pars plana vitrectomy without air-fluid exchange as controls.

We excluded patients who were younger than 20 years old at the time of surgery, had an axial length greater than 28.5 mm, IOP exceeding 30 mmHg, were unable to be followed up for at least one month, and AS-OCT could not be obtained. For patients treated in both eyes, only the first eye was included in the analyses.

This study adhered to the principles of the Declaration of Helsinki and the protocol was approved by the Institutional Review Board of Hiroshima University (E2023-0131). The requirement for informed consent was waived due to the retrospective nature of the study.

Objectives and data collection

The primary objective was to investigate the incidence of CCD following intrascleral suturing using the Yamane technique and compare it with that occurring after other pars plana vitrectomy procedures.

The data collected comprised: age at surgery, sex, slit-lamp examination, IOP measurements, best-corrected visual acuity (BCVA), preoperative axial length, central corneal thickness, and AS-OCT results. The axial length was measured using an IOLmaster (Carl Zeiss Meditec, Dublin, CA, USA), and the central corneal thickness was examined using Topcon SP-3000 (Topcon Corporation, Tokyo, Japan). AS-OCT scans were conducted using CASIA2 (Tomey, Nagoya, Japan).

Surgical procedure

A 25-gauge pars plana vitrectomy was performed using the Constellation Vision System (Alcon Laboratories, Inc., Fort Worth, Texas, USA). Cataract extraction was performed on the dislocated lens. The dislocated IOL was extruded through a 3 mm or 4 x 2 mm L-shaped scleral keratotomy.

A 30-gauge thin-walled needle (TSK ultrafine-diameter needle; Tochigi Seiko, Tochigi, Japan) was used to make two sclerotomies angled from the conjunctiva 2 mm from the corneal limbus.

Three-piece intraocular lenses (X-70; Santen, Osaka, Japan and PN6A lenses; Kowa, Tokyo, Japan) were inserted into the anterior chamber using an injector. The trailing haptic was kept outward to prevent the IOL from falling into the vitreous cavity.

The first haptic was passed through the lumen of the needle using forceps and the second haptic was passed through the lumen of the second needle using forceps. Both haptics were placed on the conjunctiva using the double-needle technique. Both ends of the haptics were cauterized using an ophthalmic cautery (Accu-Temp Cautery; Beaver Visitec, Waltham, MA, USA). The flange was fixed to the scleral tunnel. Peripheral iridotomy was performed using a vitreous surgical cutter to avoid iris capture by the intraocular lens.

AS-OCT

**Figure 1:**
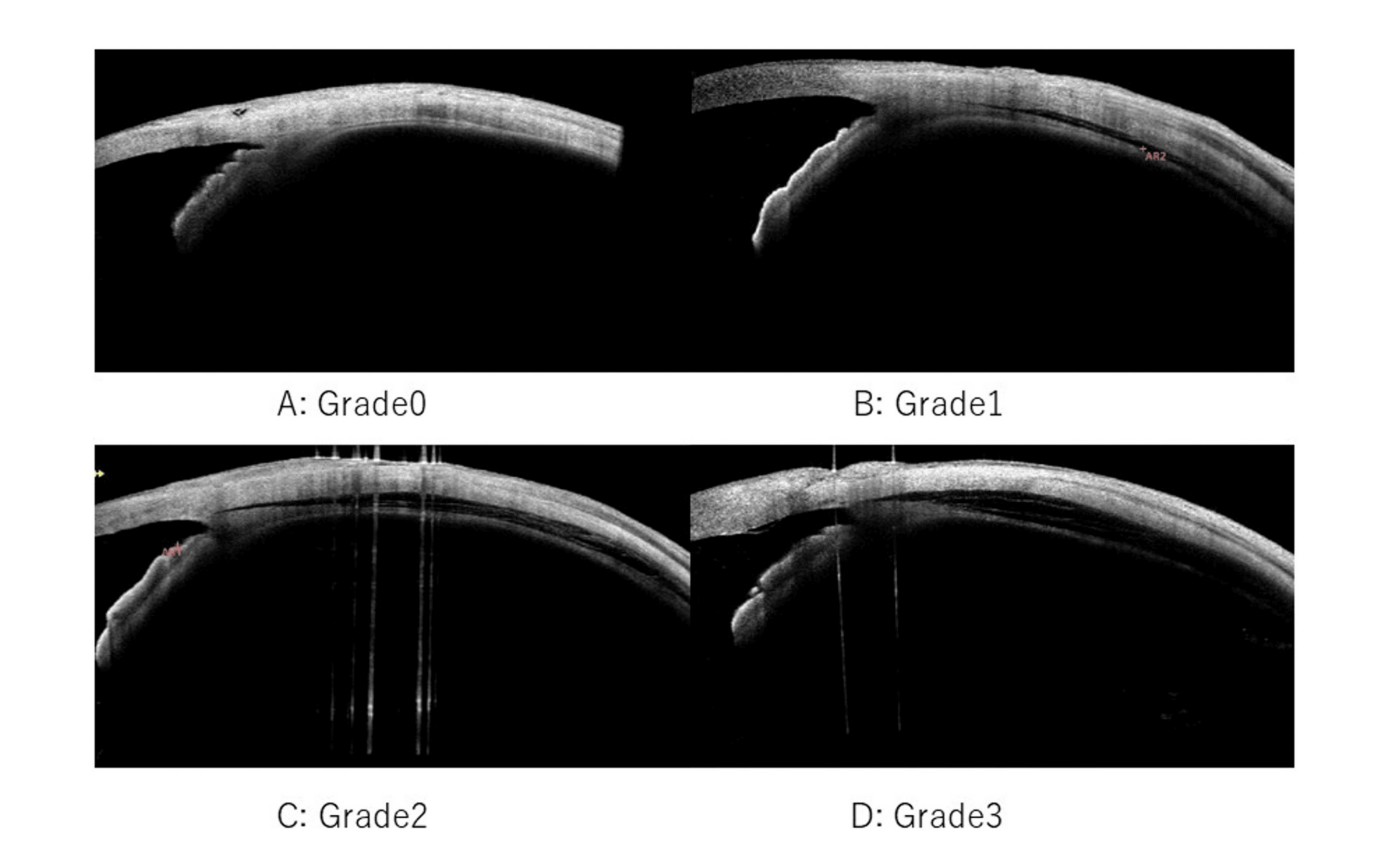
Classifications of ciliochoroidal detachment A: Grade 0 (no sign of CCD), B: Grade 1: slit-like, with CCD less than half the ciliary body thickness; C: Grade 2: band-like, with CCD at least half the ciliary body thickness; and D: Grade 3: obvious, with CCD greater than the ciliary body thickness). CCD: ciliochoroidal detachment.

AS-OCT was used for the postoperative CCD examinations. Eight directions were examined: superior, inferior, temporal, nasal, supranasal, subnasal, supratemporal, and subtemporal (Figure 1). The CCD severity classification was based on the maximum CCD value observed in the AS-OCT images, according to Miyako et al. (Figure 1) [7].

Statistical analyses

Two-tailed tests for the difference in population means or population proportions were performed using JMP PRO, version 17 (SAS Inc., Cary, NC, USA). Comparisons of the population means between the intrascleral intraocular lens fixation group and a control group of total CCD scores, postoperative IOPs and postoperative mean BCVAs (logMAR) were performed using Welch's t-test. On the other hand, a test for independence was used for CCDs. In particular, the χ2-test for the difference in the population proportions was performed for CCD (post one day), and Fisher's exact test was used for CCD (post one month). Note that Fisher's exact test was used if there was at least one expected frequency less than 5. To examine the uniformity of the two groups with respect to other variables, comparisons of population means of age, CCT, axial length, preoperative IOP, and preoperative BCVA were performed using Welch's t-test, and χ2-test for the difference in the population proportions was performed for gender and right/left eye. P-values <less than 0.05 were considered to indicate statistical significance.

## Results

A total of 45 patients were included in this study; there were 26 men and 19 women; the mean age at surgery was 70.8 years. Twenty-five underwent the Yamane technique and 20 patients who underwent other procedures were controls (Table 1). Among the eyes treated with the Yamane technique, 15 underwent surgery for IOL dislocation, five for lens dislocation, and five for aphakia. Among the controls, 17 eyes underwent pars plana vitrectomy for ERM, one for MH, and two for VMTS. The mean age at surgery, men/women ratio, and right/left eye ratio were not significantly different between the eyes that underwent the Yamane technique and the control eyes (Table 1).

**Table 1:** Study characteristics ERM: epiretinal membrane, IOL: intraocular lens, IOP: intraocular pressure, MH: stage 1 macular hole, SD: standard deviation, VMTS: vitreomacular traction syndrome. * Welch's t-test, ** χ2- test

	Intrascleral Intraocular Lens Fixation	Control	P-value
Number of eyes	25	20	
Age (years), meanSD	71.2±12.8	70.4±8.5	0.804*
Sex (men/women)	16/9	10/10	0.521**
Eye (right/left)	17/8	11/9	0.559**
Central corneal thickness (µm), meanSD	520.0±43.4	516.0±35.9	0.735*
Axial length (mm), meanSD	24.7±1.9	24.2±1.1	0.323*
Preoperative IOP (mmHg), meanSD	14.6±3.8	13.9±4.1	0.564*
Preoperative BCVA (logMAR), meanSD	0.21±0.28	0.23±0.32	0.812*
Diagnosis			
Aphakia	5		
IOL dislocation	15		
Subluxated lens	5		
ERM		17	
VMTS		2	
MH stage 1		1	

The population means of IOP on the day after surgery was significantly different between the intrascleral lens fixation group (8.4 mmHg) and the control group (11.5 mmHg). Moreover, the 95% confidence interval of the difference of the population means was [0.202,5.956]. Hence, the population means of IOP on the day after surgery was significantly lower in the intrascleral lens fixation group than in the control group. In the intrascleral lens fixation group, seven eyes (28%) had an IOP of 5 mmHg or less the day after surgery, compared with one eye (5%) in the control group. There was no significant difference in the population means of IOP at one week and one month (Table 2).

**Table 2:** Surgical results The mean value of intraocular pressure on postoperative day 1 was predominantly lower for the Yamane technique. BCVA: best-corrected visual acuity, CCD: ciliochoroidal detachment, IOP: intraocular pressure, SD: standard deviation. * Welch's t-test, ** χ2- test, *** Fisher's exact test

	Intrascleral Intraocular Lens Fixation	Control	P-value
CCD (post one day)n(%)	20(80%)	12(60%)	0.254**
CCD (post one month)n(%)	2(8%)	4(20%)	0.383***
Total CCD score, meanSD (one day)	11.4±10.2	9.2±10.9	0.489*
Total CCD score, meanSD (one month)	0.9±4.0	1.9±5.5	0.492*
Postoperative IOP (mmHg), meanSD (post one day)	8.4±4.3	11.5±5.0	0.037*
Postoperative IOP (mmHg), meanSD (post one week)	14.1±5.1	13.2±5.3	0.593*
Postoperative IOP (mmHg), meanSD (post one month)	14±3.1	14.6±3.5	0.558*
Postoperative BCVA (logMAR), meanSD (post one week)	0.2±0.3	0.1±0.2	0.313*
Postoperative BCVA (logMAR), meanSD (post one month)	0.06±0.2	0.06±0.1	0.948*

There was no difference in CCD incidence or CCD scores between the two groups. CCD persisted in two eyes in the intrascleral lens fixation group and four eyes in the control group at one month postoperatively.

The CCD site was not limited to the port creation and flange implantation area and appeared in many other locations.

## Discussion

In the present study, CCD appeared the day after surgery in 20 eyes (80%) of the patients who underwent intrascleral intraocular lens fixation using the Yamane technique, which was performed in combination with vitrectomy. The Yamane technique group had significantly lower than the control group for the population means of IOP on the day after surgery.

CCD is caused by the detachment of ciliary muscle fibers from the sclera [8,9]. This creates a direct channel from the anterior chamber to the suprachoroidal space, increasing uveoscleral outflow and reducing aqueous production secondary to ciliary detachment. Although often due to blunt trauma, it can also occur as a complication after cataract surgery, glaucoma surgery such as trabeculectomy or trabeculotomy [10,11], and after vitrectomy or panretinal photocoagulation [12]. Chen et al. studied CCD after various vitrectomies and reported that 25 of 109 eyes (54.3%) developed CCD within one week postoperatively [13].

Publications on CCD and postoperative hypotony after intrascleral IOL fixation using the Yamane technique have been limited to reports on its frequency as a postoperative complication and case reports. To the best of our knowledge, this was the first original study of CCD after using the Yamane technique.

Shelke et al. retrospectively reviewed 47 eyes with intrascleral IOL fixation using the modified Yamane technique and reported that one eye (2%) of the patients had hypotony with choroidal detachment, which was treated with laser therapy [14]. imaging CCD studies, CCD details, and the course of the disease were not mentioned.

Kelkar et al. examined 31 eyes for postoperative complications after the Yamane technique in a prospective study [15]. There was no mention of IOP on the first postoperative day. No cases of hypotony in the first week after surgery. In our study, the IOP normalized after one week.

Mishra et al. reported two cases of intrascleral fixation using the Yamane technique for traumatic cataracts [16]. Both cases involved cryotherapy because of CCD appearance in the IOL fixation axis due to continued hypotony after surgery. Although it is difficult to compare our results with theirs because of the gas-fluid exchange and the unknown IOP results on the first postoperative day, in our study, CCD did not significantly appear in the IOL fixation axis, and CCD appeared in all directions.

Yee et al. reported that CCD appeared after intrascleral fixation with the Yamane technique, and direct cyclopexy was performed because CCD and hypotonic maculopathy did not improve, even three months after surgery [17]. In the Yamane technique, a 30-gauge needle is inserted into the sclera parallel to the corneal limbus and is rotated towards the posterior chamber when retracting the haptics. This operation is considered to damage the ciliary body due to the formation of a scleral incision, rotational forces on the iris root, and pulling of the haptics out over the conjunctiva and across the suprachoroidal space. This is believed to result in the formation of ciliary clefts, which may cause CCD [17]. CCD decreases ciliary body function and reduces aqueous humor production in the ciliary epithelium, leading to low IOP.

Although there was a significant difference in the population means of IOP on the first postoperative day, IOP recovered to normal in all patients after one week, suggesting that postoperative IOP reduction may have been a temporary phenomenon. None of the patients in this study had macular hypotony or prolonged low IOP or underwent additional surgery, laser treatment, or other procedures to increase IOP.

CCD persisted in two eyes (8%) of the patients in the Yamane group and four eyes (20%) of those in the control group one month postoperatively. Once CCD appears, it does not resolve immediately and requires time to improve. CCD after 25G vitrectomy improved spontaneously by the time of examination one month postoperatively [18,19]. However, in a previous report, there was a case in which CCD did not improve and required vitrectomy, air exchange, and diathermy [20].

Limitations

The limitations of this study are the single-center, non-randomized design, small sample size, multiple surgeons, and short follow-up period. Additional multicenter prospective studies with larger sample sizes are needed to support the present results. Further investigations are necessary to elucidate the risk factors for CCD after intrascleral lens fixation using the Yamane technique.

## Conclusions

There was no significant difference in the population proportions of CCD on the day after surgery, although the rate of CCD for the intrascleral fixation group was higher, and the population mean of IOP was significantly lower than that for the control group. One week after the procedure, the IOP in the intrascleral fixation group normalized.
